# Clinical outcomes of arch expansion with Invisalign: a systematic review

**DOI:** 10.1186/s12903-023-03302-6

**Published:** 2023-08-24

**Authors:** Songyang Ma, Yunji Wang

**Affiliations:** 1 Department of Orthodontics, Stomatological Hospital of Chongqing Medical University, Chongqing, People’s Republic of China; 2 Chongqing Key Laboratory of Oral Diseases and Biomedical Sciences, Chongqing, People’s Republic of China; 3 Chongqing Municipal Key Laboratory of Oral Biomedical Engineering of Higher Education, Chongqing, People’s Republic of China

**Keywords:** Invisalign, Aligner, Expansion, Efficacy, Predictability

## Abstract

**Objective:**

This study aims to assess the scientific evidence regarding the clinical outcomes of Invisalign therapy in controlling orthodontic tooth movement.

**Materials and methods:**

An electronic search was conducted on PubMed, Cochrane Library, Web of Science, Embase, and Scopus from November 2015 to November 2022 to identify relevant articles. Methodological shortcomings were highlighted, and an evaluation of the quality of the included studies was completed using the Risk of Bias in Non-randomized Studies of Interventions (ROBINS-I) tool.

**Results:**

Fifteen non-randomized controlled trials were included in the analysis. Most non-randomized controlled trials (*n=*11; 73%) were rated with a moderate risk of bias according to the ROBINS-I tool. There were statistically significant differences between the pretreatment and posttreatment arches. The average expansion was significantly different from that predicted for each type of tooth in both the maxilla and mandible. Furthermore, the efficiency decreased from the anterior area to the posterior area in the upper arch.

**Conclusion:**

Despite the fact that arch expansion with Invisalign® is not entirely predictable, clear aligner treatment is a viable option for addressing dentition crowding. The efficacy of expansion is greatest in the premolar area. More research focusing on treatment outcomes with different materials of aligners should be conducted in the future. Overcorrection should be considered when planning arch expansion with Invisalign. In the maxilla, the expansion rate decreases from the anterior to the posterior, and presetting sufficient buccal root torque of posterior teeth may result in improved efficiency of expansion.

**Supplementary Information:**

The online version contains supplementary material available at 10.1186/s12903-023-03302-6.

## Introduction

In recent decades, orthodontic technology has made continual progress. Since Clear Aligner Therapy (CAT) was introduced in 1997, it has become an important option in orthodontic treatment [1]. Compared with traditional fixed orthodontic appliances, CAT has certain advantages, including fewer clinical emergencies, better aesthetic effect, more comfort, improved periodontal health and reduced irritation to soft tissues [2]. Now, CAT is an increasingly common orthodontic treatment option [3, 4].

Expanding the dental arch is one way to solve transverse problems and depending on the degree of maxillary compression, clinicians could choose different expansion methods such as dentoalveolar expansion or jaw expansion [5, 6]. Aligners, one common orthodontic therapy, can effectively expand the dental arch, reduce dental crowding and help achieve orthodontic treatment goals [7]. Aligners have acceptable efficiency in arch expansion both in permanent and mixed dentitions and have been recommended for selected cases with mild to moderate malocclusion [8–11]. However, aligners do not display the same accuracy as traditional fixed orthodontic appliances when it comes to transverse arch expansion [12, 13]. Moreover, although some studies have focused on improving the arch expansion effects of aligners [5, 9], traditional orthodontic compliance still falls short on beauty, oral hygiene and mucosal health [14, 15]. In this regard and to further optimize arch expansion effects and overcome the shortcomings associated with traditional orthodontic compliance, Invisalign was designed for use in clinical practice.

Recently, in order to increase the indications and efficiency of Invisalign, diversification and evolution of its primary characteristics (including material, gingival margin design, attachments, divots and auxiliaries) have been done and combined with the application of digital technology [16–18]. For instance, ClinCheck software, based on the crown of teeth, is used to analyze the efficiency and accuracy of tooth movement and can simulate dentition models before or after treatment and facilitate measurement [19]. With the benefit of gingival margin design and the absence of toxicity, the health of periodontal tissue could be well protected while using aligners [2, 15] , allowing the effects of treatment to last for a long time.

The most accurate type of tooth movement produced by aligners is the buccolingual tipping movement. This movement is achieved because the materials of the appliance are mainly bent along the buccolingual direction, which fully aligns with the logical mechanics of tooth movement [20]. Therefore, the use of software such as ClinCheck allows a precise design of Invisalign and makes the arch expansion effective [20].

There has been a lack of systematic analysis regarding the arch expansion effects of Invisalign for in nearly five years. Despite the presence of a body of literature pertaining to clear treatment, its clinical performance has not been analyzed thoroughly and a synthesis of the results remains vague. Five systematic reviews of the clinical outcomes of clear aligners exist in the literature. These reviews are not only focused on the oblique movement of the teeth but also on other types of tooth movement during treatment [1, 21–24].

However, there are only a few studies on the efficiency of arch expansion with aligners. Therefore, the purpose of the present review is to systematically search the literature and summarize the currently available scientific evidence regarding the effectiveness of arch expansion using the Invisalign system.

## Materials and methods

We conducted this systematic review following the Preferred Reporting Items for Systematic Reviews and Meta-Analyses (PRISMA) guidelines. The PRISMA checklist can be found in Additional File 1. Moreover, the protocol for this systematic review was registered in PROSPERO 2023 (Registration number: CRD42023420285).

### Eligibility criteria

The inclusion and exclusion criteria for this review are as follows:

#### Types of studies

Both prospective and retrospective studies were considered eligible for inclusion in this review. These studies were concerned with the outcomes of arch expansion with Invisalign. Only studies published in English were included in the review.

#### Participants

Only orthodontic adult patients with permanent dentition and who have expanded the dental arch after Invisalign therapy were included in this review.

#### Interventions

Studies using Invisalign therapy to expand the dental arch were included in this review. All other aligner systems were excluded.

#### Comparison group

The control method used in most relevant studies was self-control, which compares the patients’ conditions before and after treatment .

#### Outcome

The review encompassed the evaluation of any effect on clinical efficiency, predictability of ClinCheck, treatment outcomes and movement accuracy after arch expansion. Studies that evaluated arch width on actual and virtual models were included in the review.

### Exclusion criteria

The following criteria were used to exclude studies: studies older than 15 years, patients with mixed dentition, studies written in a language other than English, animal studies, case reports and studies that did not provide data and reviews of literature.

### Information sources, search strategy, study selection and data collection

An electronic search was conducted on PubMed, Cochrane Library, Web of Science, Embase and Scopus. The search was performed until November 30, 2022. An additional manual search of references in the included studies was also conducted. We used the following search term combinations: ((((aligner[Title/Abstract]) OR (invisalign system[Title/Abstract])) OR (invisalign[Title/Abstract])) OR (orthodontic appliances, removable[Title/Abstract])) AND ((expansion[Title/Abstract]) OR (arch development[Title/Abstract])).

#### Selection of studies

An initial screening of titles and abstracts was conducted independently by two researchers, who then cross-checked and reviewed the text in full to decide whether the studies were eligible. Disagreements were resolved through discussion and, when necessary, by seeking the opinion of a third researcher.

#### Data collection process

Data collection was conducted independently by two researchers followed by a discussion in order to determine the eligibility of the studies to be addressed.

### Risk of bias (RoB) assessment and effect measurement

RoB assessment was done by two independent researchers using the ROBINS-I tool. The checklist included the following three main domains of bias: preintervention, intervention and postintervention. The RoB was judged for each domain and an overall evaluation was made, categorizing RoB as low, moderate, serious, critical or having no information. The main methods used to measure effects was the mean difference.

## Results

### Study selection

**Fig. 1:**
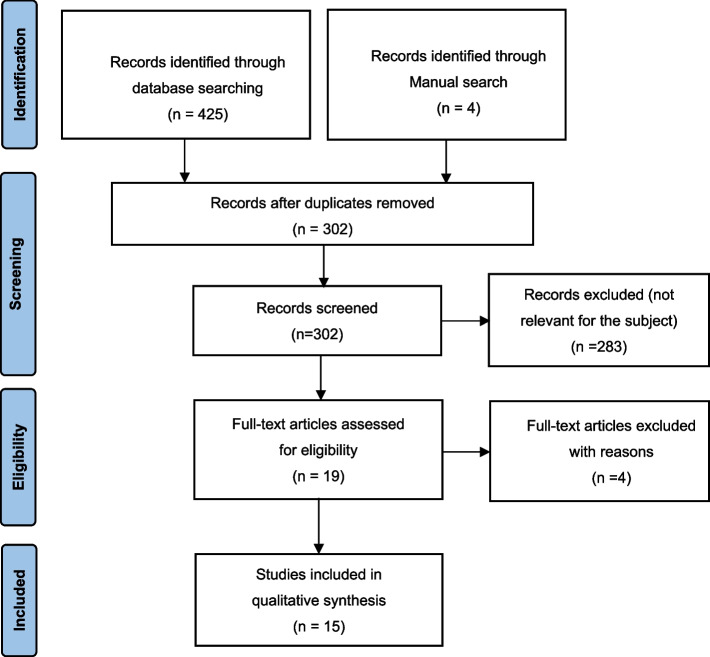
Flow diagram of study selection

An independent search was performed by two of the authors (Ma and Wang). The study selection procedure comprised of title-reading, abstract-reading and full-text-reading stages. After excluding studies that were not eligible, a full report of publications considered eligible for inclusion by either author was obtained and assessed independently. Finally, 15 articles were included in the analysis (Fig. 1).

### Characteristics of included studies

Data collection forms were used to record the desired information. The following data were collected: title, year of publication, names of authors, study design, number of participants, type of intervention, comparative groups, clinical outcomes and conclusions (Table 1).

**Table 1:** Characteristics of included studies

Author	Title	Type	Population	Intervention	Comparison	Results	Conclusion
Duncan et al. [25] (2016)	Changes in mandibular incisor position and arch form resulting from Invisalign correction of the crowded dentition treated nonextraction	Retrospective	61 adult patients (17M, 41F)	Nonextraction with or without IPR using only Invisalign	Pretreatment and posttreatment records were compared	Statistically significant increase in buccal arch: (1) Mild crowding group: intercanine (1.29mm), interpremolar (1.58mm), intermolar (1.65mm); (2) Moderate crowding group: intercanine (1.77mm), interpremolar (2.52mm), intermolar (1.87mm); (3) Sever crowding group: intercanine (1.74mm), interpremolar (3.20mm), intermolar (2.65mm)	Buccal arch expansion and IPR are important clinical tools when treating crowded dentition; intercanine, interpremolar and intermolar widths are not significant factors in the mild, moderate and severe cases
Grünheid et al. [26] (2017)	How accurate is Invisalign in nonextraction cases? Are predicted tooth positions achieved?	Retrospective	30 patients (17 F, 17 M; age 21.6±9.8 years)	Nonextraction Invisalign treatment; IPR	Comparison between posttreatment and virtual posttreatment models	Facial-lingual distance relative to the predicted position: (1) Upper arch from central incisor to second molar: -0.45mm, 0.01mm, 0.11mm, 0.15mm, 0.20mm, 0.23mm, 0.30mm (2) Lower arch from central incisor to second molar: 0.11mm, 0.01mm, 0.26mm, 0.05mm, 0.09mm, -0.08mm, -0.17mm. All tooth types showed statistically significant differences.	Invisalign can achieve predicted tooth positions with high accuracy in nonextraction cases. Maxillary arch expansion may not be fully achieved
Houle et al. [27] (2017)	The predictability of transverse changes with Invisalign	Retrospective	64 patients (41F,23M); mean age: 31.2 years (18 to 61)	Arch expansion with invisalign	Posttreatment and predicted models were compared	Statistically significant differences were found from canine to first molar: (1). Upper arch: 0.22mm, -0.58mm, 0.75mm, 0.77mm (2). Lower arch: -0.08mm, 0.07mm, 0.07mm, 0.03mm. In upper arch :72.8% accurate overall,82.9% at the cusp tips, and 62.7% at the gingival margins; in upper, arch, ClinCheck accuracy decreases when moving posteriorly into the arch. The lower arch presented an overall accuracy of 87.7%,98.9% at the cusp tips and 76.4% at the gingival	Careful planning with overcorrection and other auxiliary methods of expansion may help reduce the rate of midcourse corrections and refinements, especially in the posterior region of the maxilla
Solano- Mendoza et al. [28] (2017)	How effective is the Invisalign system in expansion movement with Ex30' aligners?	Retrospective	116 patients (73F,43M); mean age: 36.57±11.53 years	Ex30 aligner material; expansion of the posterior upper teeth	Comparisons between the actual and the planned models	Statistically significant differences from canine to first molar: (1) Cuspid width in upper arch: -0.40mm, -1.07mm, -0.80mm, -1.32mm (2) Cuspid width in lower arch: -0.68mm, -1.64mm, -1.20mm, -1.82mm. Mean difference in gingival width: (1) upper arch: canine(-0.36mm), second premolar(-0.09mm), first molar(-1.20mm) (2) Lower arch: canine(-1.39mm), second premolar(-1.28mm), first molar(-1.58mm)	The expansion planned by ClinCheck was not predictable on all varibles of gingival and cuspid width
Zhao et al. [29] (2017)	Maxillary expansion efficiency with clear aligner and its possible influencing factors	Retrospective	31 patients	Maxillary expansion with Invisalign	Comparison between the pretreatment, posttreatment and virtual posttreatment models	The increases of upper arch width from canine to second molar: 2.0mm, 2.8mm, 3.0mm, 1.8mm and 0.5 mm, with their efficiency of 68%, 70%, 68%, 55% and 29%, respectively. The mean difference of arch width between ClinCheck and posttreatment models from canine to 2nd molar: 0.80mm, 1.22mm, 1.52mm, 1.66mm, 1.85 mm. The posterior teeth showed significantly more buccal inclination than the planned position	The expansion of maxillary arch was achieved by the buccal movement of the posterior teeth with limited buccal inclination. The efficiency of expansion declines from first premolars to second molars. The planned inter-molar width had a significant influence on the efficiency of premolar expansion
Deregibus et al. [30] (2020)	Morphometric analysis of dental arch form changes in class II patients treated with clear aligners	Retrospective	27 patients (19F,8M)	Aligners; upper molars distalization; attachments, and class II elastics	Maxillary and mandibular models were compared	Regarding dimensions, significant differences between T0 and T2 from canine to first molar: (1) upper arch: 1.2mm, 2.6mm, 3.6mm, 2.6mm (2) lower arch: 1mm, 1.4mm, 2.5mm, 1.7mm.	Invisalign class II treatment results in a significant increase in arch width at the molar and premolar level in both arches. The mandibular intercanine distance did not undergo any significant change. This orthodontic approach might produce functional and stable outcomes
Haouili et al. [20] (2020)	Has Invisalign improved? A prospective follow-up study on the efficacy of tooth movement with Invisalign	Prospective	38 patients	Treated with Invisalign	Comparison between posttreatment and virtual posttreatment models	The mean accuracy of Invisalign for all tooth movements was 50%. The highest overall accuracy was achieved with a buccal-lingual crown tip (56%)	There was a marked improvement in the overall accuracy.
Morales- Burruezo et al. [31] (2020)	Arch expansion with the Invisalign system: Efficacy and predictability	Retrospective	114 adult patients (18-75 years old)	Maxillary expansion with SmartTrack aligners; intermaxillary elastics without distalization or mesialization of the dental arches	The initial models, the predicted models and the actual models were compared	Mean differences: (1) Between posttreatment and pretreatment models from canine to second molar: 1.87mm, 3.14mm, 3.45mm, 2.57mm, 0.45mm; (2) Between posttreatment and ClinChech models from canine to second molar: 0.63mm, 0.77mm, 0.81mm, 0.69mm, 0.25mm. The widths obtained the same efficacy between no crossbite group and crossbite group.	Aligners are effective for producing arch expansion, being more effective in premolar area and less effective in canine and second molar area. Overcorrection should be considered at the virtual planning stage
Zhou et al. [32] (2020)	Efficiency of upper arch expansion with the Invisalign system	Retrospective	20 patients (15F,5M); 28.5±6.3 years old	Arch expansion with Invisalign system; nonexraction; without IPR	Pre- and posttreatment models were compared	Mean differences between designed and achieved expansion from canine to first molar: 0.33mm, 0.53mm, 0.65mm, 0.74mm. The efficiencies of crown expansion movement for the canine, first premolar, second premolar, and first molar were 79.75%, 76.10%, 73.27%, and 68.31%, respectively. No significant change was observed in maxillary basal bone width. The preset expansion amount and initial maxillary first molar torque were significantly negatively correlated with efficiency of bodily expansion movement	The Invisalign system can increase arch width by tipping movement of posterior teeth. The efficiency of bodily buccal expansion for maxillary first molars averaged 36.35%. It's necessary to preset sufficient buccal root torque of posterior teeth according to the preset amount of expansion and initial torque
Lione et al. [33] (2021)	Maxillary arch development with Invisalign system	Prospective	28 patients (16F,12M); mean age 31.9±5.4 years	Nonextraction; invisalign system; Invisalign attachments	Pretreatment, predicted, and posttreatment models were compared	Mean differences between pretreatment and posttreatment values: canine(2.2mm), first premolar(3.5mm), second premolar(3.8mm), first molar (mesial cusps, distal cusps, transpalatal: 2.6mm, 2.2mm, 1.6mm), second molar (mesial cusps, distal cusps, transpalatal: 0.7mm, 0.3mm, 0.5mm). Mean differences between ClinCheck and posttreatment values: canine(1.6mm), first premolar(0.3mm), second premolar(0.3mm), first molar (mesial cusps, distal cusps, transpalatal: -0.2mm, 0.4mm, 0.3mm), second molar (mesial cusps, distal cusps, transpalatal: -0.1mm, 0.6mm, 1.2mm). The greatest increase of maxillary width was detected at the upper first and second premolars. A decreasing expansion gradient was observed moving from the anterior to posterior part of the arch.	A progressive reduction in the expansion rate from the canine to the posterior regions, with the greatest net increase at the premolars. The Invisalign system can increase arch width by increasing the buccal tipping of maxillary teeth.
Riede et al. [34] (2021)	Maxillary expansion or contraction and occlusal contact adjustment: effectiveness of current aligner treatment	Retrospective	30 patients(23F,7M); mean age 25.7(15-43) years	Invisalign aligner treatment exclusively with the SmartTrack material; nonextraction	Comparison between the pretreatment, posttreatment and virtual posttreatment models	Mean difference was not mentioned. The median discrepancy between ClinCheck and posttreatment models from canine to first molar: (1). Level of cusp tip: 0.35mm, 0.425mm, 0.425mm, 0.475mm (2) level of gingival margins: 0.40mm, 0.375mm, 0.425mm, 0.525mm	45% effectiveness in achieving treatment objectives of transverse contraction or expansion. The effectiveness was generally not increased with SmartTrack compared to Ex30® material.
Bernardez et al. [6] (2021)	Efficacy and predictability of maxillary and mandibular expansion with the Invisalign® system	Retrospective	64 upper arches and 51 lower arches	Invisalign treatment; models without attachments	Pretreatment, predicted, and posttreatment models were compared	Mean difference between ClinCheck and posttreatment models from canine to first molar: upper arch:(1). Level of cusp tip: 0.90mm, 0.50mm, 0.50mm, 1.20mm (2) level of gingival margins: 4.70mm, 7.20mm, 8.60mm, 6.30mm Lower arch: (1) Level of cusp tip: 0.60mm, 0.20mm, 0.13mm, 0.40mm (2) level of gingival margins: 3.80mm, 4.90mm, 5.90mm, 4.66mm	The Invisalign system aligners (SmartTrack material) offer high degree of predictability. The most predictable level of expansion was moderate, having being the lower arch more foreseeable at the gingival level than the upper arch.
Goh et al. [35] (2022)	The predictability of the mandibular curve of Wilson, buccolingual crown inclination, and transverse expansion expression with Invisalign treatment	Retrospective	42 patients	Invisalign aligners without intermaxillary elastics, bite ramps, or auxiliaries. Nonextraction	Comparison between posttreatment and virtual posttreatment models	Mean differences between posttreatment and predicted lower arch models from canine to second molar: -0.48mm, -0.39mm, -0.62mm, -0.11mm, 0.68mm. The first molars encountered 0.52 mm more buccal crown inclination. No other teeth experienced statistically significant buccolingual inclination differences. Only the second molars experienced significantly more arch expansion	Only the mandibular second molars experienced more expansion than ClinCheck
Lione et al. [36] (2022)	Analysis of Maxillary First Molar Derotation with Invisalign Clear Aligners in Permanent Dentition	Prospective	40 patients (20 F, 20M);22.4 *±* 3.9 years	Class II patients treated with clear aligners; nonextraction	The pretreatment, posttreatment and final ClinCheck model were compared	The mean transversal expansion of the maxillary first molar: (1) on the pretreatment and posttreatment models: the mesial buccal cusps (2.2mm), the distal buccal cusps(1.5mm) (2) on the ClinCheck and posttreatment models: the mesial buccal cusps(0.1mm), the distal buccal cusps(0.11mm). No statistically significant changes between the ClinCheck model and the posttreatment model.	Distal rotation of maxillary first molars involves the expansion and distal shift of buccal cusps. Aligners are effective at allowing maxillary distal molar rotation. Transverse expansion shows high accuracy
Tien et al. [37] (2022)	The predictability of transverse changes with Invisalign	Retrospective	57 adult patients	Planned expansion of at least 3 mm with Invisalign	Pretreatment, predicted, and posttreatment models were compared	Mean differences of expansion from canine to second molar between predicted and posttreatment models: (1) upper arch: 0.75mm, 0.82mm, 0.83mm, 1.32mm, 2.11mm (2) lower arch: 0.48mm, 0.28mm, 0.54mm, 0.76mm, 2mm. The average expansion was significantly different from that predicted for each type of tooth in both the maxilla and mandible	The amount of predicted expansion is not achieved and varies according to tooth type and arch. Discretion is required when compensating for inaccuracy

*F* Female, *M* Male, *IPR* Interproximal reduction

### Quality assessment

To determine the methodological quality and level of evidence, the classification system described by the Swedish Council on Technology Assessment in Health Care was used [38]. Table 2 provides the criteria used to judge each study.

**Table 2:** Swedish council on technology assessment in health care criteria for grading assessed studies

Grade A	Grade B	Grade C
High value of evidence	Moderate value of evidence	Low value of evidence
All criteria should be met:	All criteria should be met:	One or more of the conditions below:
-Randomized clinical study or a prospective study with a well defined control group -Defined diagnosis and endpoints -Diagnostic reliability tests and reproducibility tests described -Blinded outcome assessment	-Cohort study or retrospective case series with defined control or reference group -Defined diagnosis and endpoints -Diagnostic reliability tests and reproducibility tests described	-Large attrition -Unclear diagnosis and endpoints -Poorly defined patient material

The definitions of evidence level are presented in Table 3. The methodological quality was moderate for 12 of the included studies [6, 20, 25–29, 31–33, 35, 37] and limited for the remaining 3 studies [30, 34, 36] , as shown in Table 4.

**Table 3:** Definitions of evidence level

Level	Evidence	Definition
1	Strong	At least two studies assessed with level “A”
2	Moderate	One study with level “A” and at least two studies with level “B”
3	Limited	At least two studies with level “B”
4	Inconclusive	Fewer than two studies with level “B”

**Table 4:** Grading of Included Studies

Author, year	Grade
Duncan et al. (2016) [25]	B
Grünheid et al. (2017) [26]	B
Houle et al. (2017) [27]	B
Solano-Mendoza et al. (2017) [28]	B
Zhao et al. (2017) [29]	B
Haouili et al. (2020) [20]	B
Deregibus et al. (2020) [30]	C
Morales-Burruezo et al. (2020) [31]	B
Zhou et al. (2020) [29]	B
Lione et al. (2021) [33]	B
Riede et al. (2021) [34]	C
Bernardez et al. (2021) [6]	B
Goh et al. (2022) [35]	B
Lione et al. (2022) [36]	C
Tien et al. (2022) [37]	B

Therefore, conclusions obtained from this review were based on a limited level of evidence. The most recurrent sources of bias were related to the study type and the lack of blinded outcome assessment. However, only two retrospective studies [30, 34] and one prospective study [36] were rated as having evidence of low value. Furthermore, the reason why they were rated as low was the lack of reproducibility tests or well-defined patient materials.

Finally, we also used the ROBINS-I tool for RoB assessment to evaluate the bias of included studies in Table 5. Among the included studies, 2 [30, 34] had a serious RoB and 2 [20, 29] of the remaining 13 had a critical RoB (Table 5). The most recurrent sources of bias were related to the selection of patients and the lack of blinded outcome assessment. Besides, because the comparison was between the patient’s post-treatment models and “their own” predicted models, the RoB of confounding was low. However, Zhao’s study [29] and Haouili’s [20] study showed a critical RoB due to the lack of well-defined patient material. In addition, the patient’s age range was relatively large in Riede’s study [34]. In contrast, other studies selected patients with a smaller age range, which could affect the predictability of arch expansion. Most authors chose the cuspid of a tooth as the landmark for measurements, but Grünheid [26] and Zhao [29] did not. Furthermore, Zhao did not perform reliability testing [29]. Overall, the bias in measuring outcomes was serious.

**Table 5:** Risk of bias of included studies by robins‐i quality assessment scale

	Domains							
	Preintervention		Intervention	Postintervention				
Author	Risk of Bias of Confounding	Risk of Bias in the Selection of Participants	Risk of Bias in the Intervention Classification	Risk of Bias as a Result of Deviation From Planned Intervention	Risk of Bias as a Result of Missing Data	Risk of Bias in the Measurement of Results	Risk of Bias in the Selection of Reported Results	General Judgment of Risk of Bias
Duncan et al. [25] (2016)	Low	Moderate	Low	Low	Low	Low	Low	Moderate
Grünheid et al. [26] (2017)	Low	Moderate	Low	Low	Low	Moderate	Low	Moderate
Houle et al. [27] (2017)	Low	Moderate	Low	Low	Low	Low	Low	Moderate
Solano- Mendoza et al. [28] (2017)	Low	Moderate	Low	Moderate	Low	Moderate	Low	Moderate
Zhao et al. [29] (2017)	Low	Critical	Low	Low	Low	Serious	Moderate	Critical
Deregibus et al. [30] (2020)	Low	Moderate	Low	Low	Low	Serious	Low	Serious
Haouili et al. [20] (2020)	Low	Critical	Low	Low	Low	Moderate	Low	Critical
Morales- Burruezo et al. [31] (2020)	Low	Moderate	Low	Low	Low	Moderate	Low	Moderate
Zhou et al. [32] (2020)	Low	Moderate	Low	Low	Low	Low	Low	Moderate
Lione et al. [33](2021)	Low	Moderate	Low	Low	Low	Low	Low	Moderate
Riede et al. [34](2021)	Low	Serious	Low	Low	Low	Low	Low	Serious
Bernardez et al. [6] (2021)	Low	Moderate	Low	Moderate	Low	Low	Low	Moderate
Goh et al. [35](2022)	Low	Moderate	Low	Low	Low	Low	Low	Moderate
Lione et al. [36](2022)	Low	Moderate	Low	Low	Low	Moderate	Low	Moderate
Tien et al. [37](2022)	Low	Moderate	Low	Moderate	Low	Low	Moderate	Moderate

### Clinical findings

#### Efficacy of expansion

In all the included studies, the efficacy of arch expansion with Invisalign could be evaluated by comparing the pretreatment and posttreatment models. It is clear that there were statistically significant differences between the pretreatment and posttreatment arches, indicating that Invisalign effectively expanded the dental arch [6, 19, 20, 28–34, 36, 37]. Deregibus concluded that Invisalign class II treatment resulted in a significant increase in arch width at the molar and premolar levels in both arches [30]. However, in Morales-Burruezo’s study, expansion was more effective in the premolar area and less effective in the canine and second molar areas [31]. Furthermore, the efficacy of expansion was different between the upper and lower arches [6].

#### Predictability of expansion

The predictability of expansion, which refers to the ability to predict final outcomes at the beginning of Invisalign treatment, could be examined by comparing the difference between the virtual posttreatment digital model simulated on the ClinCheck software and the actual digital model obtained by scanning the posttreatment model. The predictability of expansion is also called the efficiency or the accuracy of arch expansion. Among the included studies, 13 of them focused on the predictability of arch expansion with Invisalign [6, 20, 26–34, 36, 37]. The average expansion was significantly different from that predicted for each type of tooth in both the maxilla and mandible, and both underexpansion and overexpansion were observed [26]. However, no statistically significant changes between the ClinCheck model and the posttreatment model were discovered in Lione’s study [36]. Notably, in Zhou’s study, the efficacy of crown expansion movement in the upper arch for the canine, first premolar, second premolar and first molar were 79.75%, 76.10%, 73.27% and 68.31%, respectively [32]. Clearly, the efficiency decreased from the anterior area to the posterior area in the upper arch, which was similar to Lione’s finding [33].

#### Types of materials

Invisalign appliances made of different materials were used to investigate whether there was a difference in the predictability of dental arch development. According to Riede, the effectiveness of achieving transverse values as planned was generally not increased with the use of SmartTrack compared to the previously used Ex30 material [34]. However, the Invisalign system aligners (SmartTrack material) offered a high degree of predictability both in the upper and lower arches in Bernardez’s study [6]. Furthermore, during orthodontic treatment with Ex30 aligners, the predictability of expansion depending on the magnitude of the planned expansion was not predictable, while canine depth, arch depth, molar inclination and molar rotation were shown to be predictable [28].

#### Other findings

When treating crowded dentition, buccal arch expansion and interproximal reduction are important clinical tools [25]. Besides, careful planning, including overcorrection and the use of other auxiliary methods of expansion, should be taken into consideration, which will result in a reduction in the rate of midcourse corrections and refinements [27]. The Invisalign system can increase arch width by the tipping movement of posterior teeth, and no significant change was observed in maxillary basal bone width [32]. Furthermore, the amount of preset expansion amount and initial maxillary first molar torque were significantly negatively correlated with the efficiency of bodily expansion movement [32].

## Discussion

Most of the collected literature, including that for which full text cannot be obtained, was published in the past three years, indicating a trend in which dental arch expansion has been a focus of research on clear treatment. Six systematic reviews on Invisalign are currently available [1, 21–24, 39] and three of them evaluate the efficiency of arch expansion [1, 23, 24]. However, these reviews have not paid great attention to the changes in the transverse dimension, and they relied on studies published prior to ours. Therefore, we decided to submit a comparatively precise and innovative systematic review to assist in clinical practice.

This review included 12 retrospective studies and 3 prospective studies. After an assessment of the quality of the included studies, limited conclusions were drawn because there were more than two studies having a grading of B. Besides the limited sample size, these 15 studies were drawn from different countries and regions and have different inclusion and exclusion criteria for subjects, which increases the bias of this systematic review. Moreover, the age differences of the patients included in each study would also influence the results. There is also a lack of studies published in authoritative journals among the included studies. In this systematic review, further mathematical analysis is needed to perform a comprehensive exploration of the clinical outcomes of arch expansion with Invisalign. The perspective and methods of interpreting data that we used were also not innovative enough. Finally, there is a lack of a multicenter study in the included studies—one will be needed in the future to clarify the clinical outcomes of arch expansion with Invisalign.

Two retrospective studies [29, 32] reported that the expansion effect of the dental arch is mainly caused by the tipping movement of teeth, which is manifested as the change of transverse width. Furthermore, Duncan et al. [25] mentioned that the arch expansion achieved by the buccal tipping movement of teeth, which is a kind of transversal movement, is one of the significant pathways to resolve dentition crowding. In Duncan’s research [25], itis noteworthy that the greatest expansion efficacy occurred in the premolar area, which is supported by Zhou et al. [32] and Morales-Burruezo et al. [31]. Furthermore, the results of one retrospective study [30] and one prospective study [36] showed a significant increase of arch width and functional and stable outcomes for patients who have undergone class II clear treatment.

Lione et al. [36] and Grünheid et al. [26] stated that although maxillary arch expansion may not be fully attained, in nonextraction cases, Invisalign is able to achieve predicted tooth positions with high accuracy. They also reported that aligners made of different materials do not have a significant difference in efficacy [34]. However, Solano-Mendoza [28] and Vidal-Bernardez [6] present a contrasting viewpoint. Among patients treated with Ex30 aligners the predictability of upper arch expansion is not predictable [28], but—on the contrary—among those treated with SmartTrack material, the predictability is shown to be high in both the upper and lower arches [6]. Therefore, more research focusing on aligner materials should be conducted.

Morales-Burruezo et al. [31], Lione et al. [33] and Tien et al. [37] stated that there were statistically significant differences between the predicted and actual treatment outcomes. Therefore, overcorrection should be considered on ClinCheck in order to obtain expected outcomes. Furthermore, discretion is required when overcorrecting to compensate for expansion inaccuracy. Notably, a progressive reduction in the expansion rate from the anterior area to the posterior region in the upper arch was observed in three retrospective studies [29, 31, 32] and one prospective study [33]. The reasons for this reduction may be differences in root anatomy and cortical bone thickness, a higher occlusal load, great soft tissue resistance in the posterior region and a decline of mechanical efficiency from the anterior to the posterior [32]. Furthermore, the amount of preset expansion and initial maxillary first molar torque are significantly negatively correlated with the efficiency of expansion movement. Thus, presetting sufficient buccal root torque of posterior teeth is an important strategy for improving the efficiency of expansion [32].

Above all, in this systematic review, we found that the use of Invisalign holds promise for orthodontic patients who undergo arch expansion treatment. In some studies, researchers have provided evidence indicating that the effects of Invisalign are superior to those of other common orthodontic treatments. Therefore, the use of Invisalign might be a suitable therapy for orthodontic patients who will undergo arch expansion. The available clinical studies have mainly concentrated on adolescents, and there is also a lack of reporting on adverse reactions associated with Invisalign. As the average age of patients receiving orthodontic treatment is gradually increasing, in order to provide patients with superior and effective orthodontic treatment, further studies are needed to address these problems .

## Conclusions

- Despite the fact that arch expansion with Invisalign® is not completely predictable, clear treatment is a viable option for resolving dentition crowding.
- The efficacy of expansion is highest in the premolar area.
- Research focusing on treatment outcomes with different materials of aligners should be conducted in the future.
- Overcorrection should be considered when planning an arch expansion with Invisalign.
- In the maxilla, the expansion rate decreases from the anterior to the posterior.
- In the maxilla, presetting sufficient buccal root torque of posterior teeth may enhance the efficiency of expansion.

### Supplementary Information

**Additional file 1. **

## Data Availability

The datasets used and/or analyzed during the current study are available from the corresponding author upon reasonable request.
